# Dynamic Handgrip Exercise: Feasibility and Physiologic Stress Response of a Potential Needle-Free Cardiac Magnetic Resonance Stress Test

**DOI:** 10.3389/fcvm.2021.755759

**Published:** 2021-11-29

**Authors:** Andreas Ochs, Michael Nippes, Janek Salatzki, Lukas D. Weberling, Johannes Riffel, Matthias Müller-Hennessen, Evangelos Giannitsis, Nael Osman, Christian Stehning, Florian André, Hugo A. Katus, Norbert Frey, Matthias G. Friedrich, Marco M. Ochs

**Affiliations:** ^1^Department of Cardiology, University of Heidelberg, Heidelberg, Germany; ^2^DZHK (German Centre for Cardiovascular Research) Partner Site, Heidelberg, Germany; ^3^Department of Radiology and Radiological Science, School of Medicine, John Hopkins University, Baltimore, MD, United States; ^4^Myocardial Solutions, Inc., Morrisville, NC, United States; ^5^Philips Healthcare, Hamburg, Germany; ^6^Departments of Medicine and Diagnostic Radiology, McGill University, Montreal, QC, Canada

**Keywords:** stress-test, handgrip, longitudinal strain, fSENC, CMR

## Abstract

**Background:** Cardiac magnetic resonance (CMR) pharmacological stress-testing is a well-established technique for detecting myocardial ischemia. Although stressors and contrast agents seem relatively safe, contraindications and side effects must be considered. Substantial costs are further limiting its applicability. Dynamic handgrip exercise (DHE) may have the potential to address these shortcomings as a physiological stressor. We therefore evaluated the feasibility and physiologic stress response of DHE in relation to pharmacological dobutamine-stimulation within the context of CMR examinations.

**Methods:** Two groups were prospectively enrolled: (I) volunteers without relevant disease and (II) patients with known CAD referred for stress-testing. A both-handed, metronome-guided DHE was performed over 2 min continuously with 80 contractions/minute by all participants, whereas dobutamine stress-testing was only performed in group (II). Short axis strain by fast-Strain-ENCoded imaging was acquired at rest, immediately after DHE and during dobutamine infusion.

**Results:** Eighty middle-aged individuals (age 56 ± 17 years, 48 men) were enrolled. DHE triggered significant positive chronotropic (HR_rest_: 68 ± 10 bpm, HR_DHE_: 91 ± 13 bpm, *p* < 0.001) and inotropic stress response (GLS_rest_: −19.4 ± 1.9%, GLS_DHE_: −20.6 ± 2.1%, *p* < 0.001). Exercise-induced increase of longitudinal strain was present in healthy volunteers and patients with CAD to the same extent, but in general more pronounced in the midventricular and apical layers (*p* < 0.01). DHE was aborted by a minor portion (7%) due to peripheral fatigue. The inotropic effect of DHE appears to be non-inferior to intermediate dobutamine-stimulation (GLS_DHE_= −19.5 ± 2.3%, GLS_Dob_= −19.1 ± 3.1%, *p* = n.s.), whereas its chronotropic effect was superior (HR_DHE_= 89 ± 14 bpm, HR_Dob_= 78 ± 15 bpm, *p* < 0.001).

**Conclusions:** DHE causes positive ino- and chronotropic effects superior to intermediate dobutamine-stimulation, suggesting a relevant increase of myocardial oxygen demand. DHE appears to be safe and timesaving with broad applicability. The data encourages further studies to determine its potential to detect obstructive CAD.

## Introduction

Cardiac magnetic resonance (CMR) stress testing to quantify myocardial ischemia represents an excellent prognostic tool that is non-inferior to invasive fractional flow reserve measurements, and is therefore suggested by current guidelines to direct revascularization therapy in chronic coronary syndrome (1–5). Current CMR protocols, however, have certain shortcomings restricting their applicability in a relevant number of patients (6–9). On one hand, there are safety concerns due to the reliance on pharmacological agents. Myocardial perfusion imaging relies on vasodilating stressors (e.g., adenosine or regadenosone) and gadolinium-based contrast agents. Furthermore, although less commonly used, adrenergic stressors are a prevalent pharmacological agent (e.g., Dobutamine) that increase the myocardial oxygen demand and allow to detect coronary insufficiencies by inducible wall motion abnormalities. Although, side effects of these agents are rare, contraindications and complicating risk factors such as chronic kidney injury, hemodialysis, or bronchial asthma for vasodilators (e.g., adenosine or regadenoson) or severely reduced ejection fraction (EF) or ventricular arrhythmogenicity for adrenergic stressors have to be critically assessed. On the other hand, the time consuming CMR stress tests cause tremendous running costs for CMR scanners and the personnel. The use of the above mentioned pharmacological agents is also coupled with incomplete reimbursement. These arguments taken together, have led to a hesitant adoption of CMR stress testing by the majority of health care providers worldwide despite its proven benefits. Various physiological exercises were sought to replace these downsides of pharmacological stressors, but neither MR-conditional ergometers, steppers, or treadmills could be established, as these protocols were found to be time-consuming, and exercise-related body motion severely affected image quality (10).

We aimed to address these shortcomings by dynamic handgrip exercise (DHE) as a modified needle-free physiological stress test. Unlike previously assessed static handgrip maneuvers, we expected a “dobutamine”-equivalent, positive ino- and chronotropic effect in response to repetitive isotonic both-handed contractions without relevant body motion (11–14). The goal of this study was to assess the feasibility and hemodynamic effect of DHE in healthy volunteers and in relation to varying doses of continuous dobutamine infusion.

## Materials and Methods

### Study Population and Design

Participants were prospectively enrolled at our Department between December 2019 and March 2020 after an individual signed consent, they were divided into one of the two groups: (I) volunteers without relevant history of disease and (II) patients with known CAD, detected by previously performed invasive coronary angiography, who were referred for CMR stress testing and underwent dobutamine stress (predominantly due to contraindications to adenosine, e.g., bronchial asthma). All participants answered a specific questionnaire for symptoms (pre- and post-stress), risk factors, and relevant preexisting illnesses. Group (I) comprised of healthy volunteers and excluded individuals with history, signs, or symptoms of a cardiac disease, except mild arterial hypertension or other existing, isolated cardiovascular risk factors. Participants unable to perform DHE, with impaired LV (left ventricular) EF < 50% patients, evidence of stress-induced perfusion deficit, or previous myocardial infarction were excluded. Five patients with CAD were finally excluded due to a positive dobutamine stress test. The study was approved by the institution's ethics committee and was in accordance to the Declaration of Helsinki.

### CMR Acquisition Protocol

**Figure 1:**
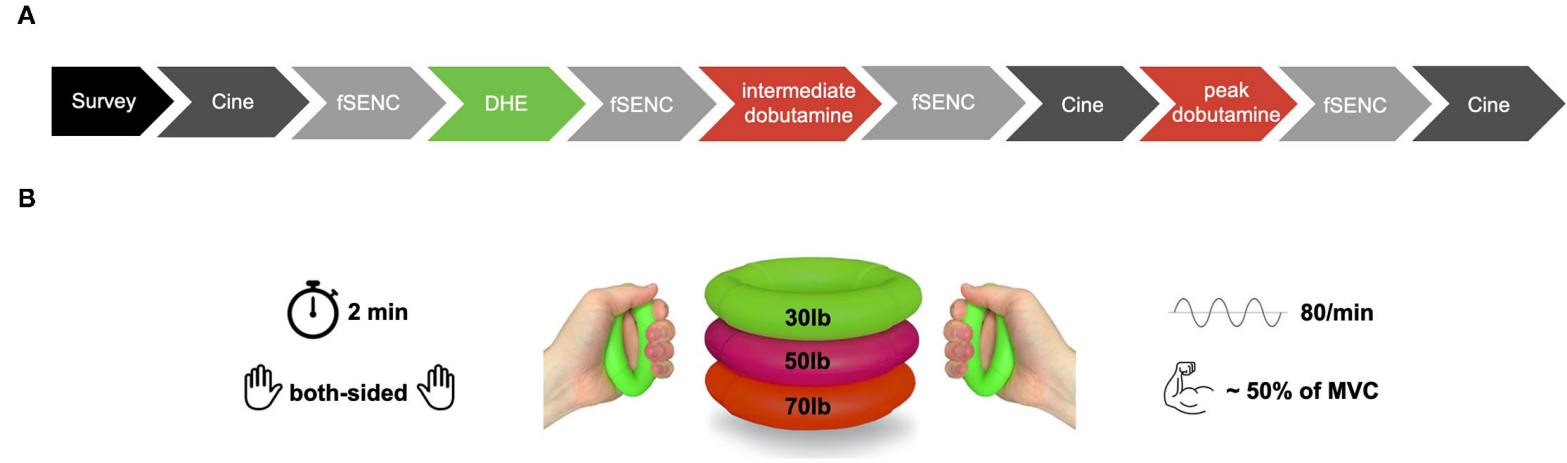
**(A)** CMR protocol of the DHE study. Additional fSENC-sequences were acquired at rest, after 2 min of DHE, and (in the group of patients with CAD) during intermediate (20 μg/kg body weight/minute) and peak dobutamine/atropine stress (40 μg/kg body weight/minute + atropine). DHE was performed after all standard sequences at rest and before the start of pharmacological stress. **(B)** Illustration of DHE. Both-sided, metronome-guided rhythmic hand contractions were performed for 2 min at a frequency of 80/min. Handgrip rubber rings at about 50% of maximal voluntary contraction (MVC) were used. CMR, cardiac magnetic resonance imaging; fSENC, fast strain-encoded magnetic resonance imaging; DHE, dynamic handgrip exercise; CAD, coronary artery disease; MVC, maximal voluntary contraction.

Cardiac magnetic resonance was performed on a 1.5-Tesla or 3-Tesla clinical scanner (Ingenia and Ingenia CX®, Philips Healthcare, Best, The Netherlands) with a dedicated 32-element cardiac-phased array receiver coil. R-wave triggered SSFP cine sequences were acquired in long- (2-, 3-, 4-chamber views) and short axis (apical, midventricular and basal) views with, 35 phases per cardiac cycle. As previously described, fSENC was performed as a single heartbeat acquisition (15). fSENC sequences were acquired at rest and after 2 min of DHE at apical, midventricular, and basal short axis layers. The specific study protocols for healthy individuals (group I) performing DHE alone was extended in patients with CAD (group II) with a dobutamine stress test, as demonstrated in Figure 1A, after DHE part. In dobutamine stress, infusion rate started at 10 μg/kg body weight/minute with increments of 10 μg/kg body weight/minute every 3 min and a maximum dose of 40 μg/kg body weight/minute. Additionally, fractions of 0.25 mg atropine were substituted to achieve the target heart rate [85% × (220-age)]. Three short axis and three long axis cine sequences were acquired at each stress level (16).

### Dynamic Handgrip Exercise

Dynamic handgrip exercise was performed with both-sided, metronome-guided rhythmic hand contractions for 2 min (Figure 1B). Commercially available, CMR-capable rubber handgrip rings in three different strengths (30, 50, and 70 lb) were offered to the subjects before the scan started. Maximal voluntary contraction (MVC) for each person was quantified by a dynamic handgrip trainer. The handgrip ring, closest to 50% of MVC, was chosen for DHE which was performed at a frequency of 80/min, acoustically indicated by a metronome beat over CMR voice communication. In case of premature physical exhaustion during the examination, patients were instructed to alert the medical personnel by pressing the alert alarm button held within the subject's hand, which subsequently led to the immediate initiation of fast Strain-ENcoded magnetic resonance imaging (fSENC). An adequate execution of DHE was supervised by the attending technician *via* visual control and an adequate heart rate (HR) response, which was controlled continuously by electrocardiogram (ECG) monitoring. DHE was stated as insufficient when hand movement rate continuously dropped below 80/min. Shortly before finishing the 2 min of handgrip exercise, the subjects were advised to hold their breath after expiration to start fSENC sequence manually.

### Image Analysis

Analysis of ventricular volumes, LV myocardial mass, and LVEF were derived from short- and long axis slices on commercially available workstations (IntelliSpace Portal®, Philips Healthcare) and a dedicated post-processing software (cvi^42^™ v5.5, Circle *Cardiovasc Imaging*, Calgary, Canada) from CMR-experienced physicians. Dobutamine stress was analyzed according to current CMR interpretation guidelines (17).

**Figure 2:**
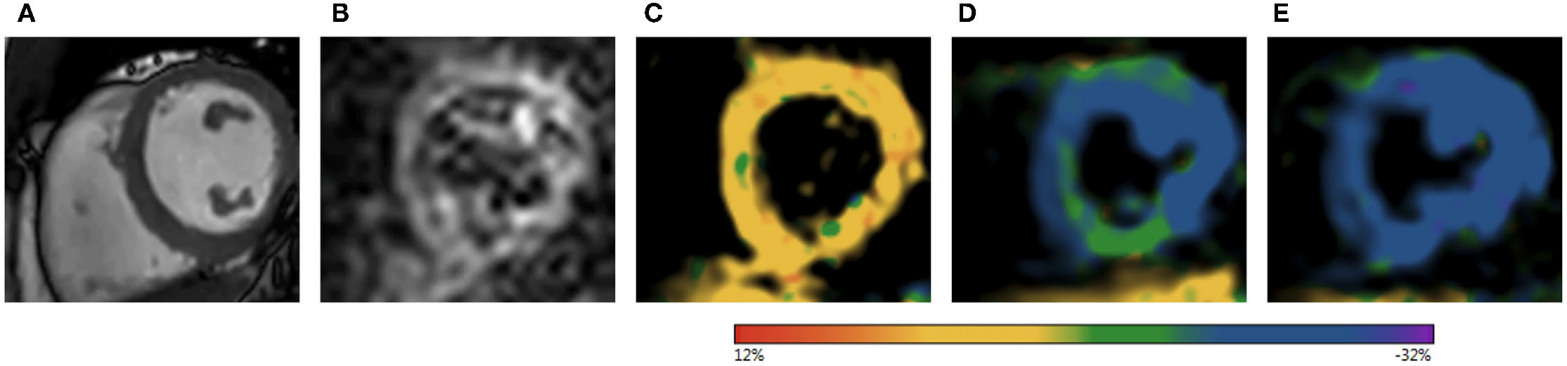
Example of the image acquisition and GLS response of a healthy volunteer as visualized in the MyoStrain® software. **(A)** Cine sequence of a midventricular short axis slice. **(B)** fSENC-images of midventricular short axis slice at end-diastole. **(C–E)** Corresponding color-coded images after post-processing at end-diastole **(C)**, end-systole at rest **(D)**, and after 2 min of DHE **(E)**. As demonstrated in **(D,E)**, peak systolic longitudinal strain increased after DHE (color scale represents regional longitudinal strain). GLS, global longitudinal strain; fSENC, fast strain-encoded magnetic resonance imaging; DHE, dynamic handgrip exercise.

For the interpretation of fSENC sequences and measurements of longitudinal strain at rest, after DHE and during dobutamine stress, a dedicated software (MyoStrain 5.2.1 Myocardial Solutions, Inc., Morrisville, North Carolina, USA) was used (Figure 2): endo- and epicardial borders were drawn manually at end-systole for each short-axis slice resulting in segmental and global longitudinal strain values. The examiners underwent specific training for the MyoStrain® software. For intra- and interobserver variability, 10 randomly selected subjects were analyzed twice. As reported before (18, 19), GLS response after DHE was classified as stable when ΔGLS ≥ −0.5% and ≤ 0.5%, increased when ΔGLS < −0.5%, and decreased when ΔGLS > 0.5%.

### Statistical Methods

A dedicated software, MedCalc™ v20.014 (MedCalc software, Mariakerke, Belgium) was used for statistical analysis. Normal distribution was assessed using Shapiro-Wilk test. Continuous parameters were expressed as mean ± standard deviation for parametric and as median with interquartile range (IQR) for non-parametric variables. For the comparison of continuous variables between two groups, Student's *t*-test and Mann–Whitney U test were used as applicable. Not normal distributed continuous variables were tested for differences using the non-parametric Wilcoxon test. Receiver operating characteristics analysis was used to prove the test accuracy for the diagnosis of CAD and to define their cut-off values. The intra- and interobserver variability was described using the intraclass correlation coefficient (ICC with 95% CI) with a two-way random model with absolute agreement. A *P*-value <0.05 was regarded as statistically significant.

## Results

### Study Population

Eighty middle-aged individuals including two groups of healthy individuals (I) and patients with CAD (II) (mean age 56 ± 17 years; 48 men) were enrolled (Table 1). All subjects were in sinus rhythm. CMR revealed regular biventricular function and morphological dimensions in all individuals (LVEF 62 ± 6%, LVEDV 145 ± 33 ml, LV mass 103 ± 28 g). Fifty-two subjects (65%) underwent the study examination on 1.5 Tesla MR scanner, the others on a 3 Tesla clinical MR scanner. Mean systolic blood pressure at rest was 124 ± 10 mmHg, diastolic blood pressure at rest was 78 ± 7 mmHg. In patients with CAD, three had mild CAD, five a single-vessel disease, and 22 a multivessel disease. Nineteen patients underwent previous percutaneous intervention of coronaries, four had prior coronary artery bypass graft surgery. Patients with CAD had a high cardiovascular risk profile: 23 had arterial hypertension, 20 hypercholesterinemia, 10 a family history of cardiovascular disease, 21 were obese, seven had diabetes, and 12 were smokers.

**Table 1:** Baseline characteristics of all individuals (*n* = 80) and divided by groups.

	**All individuals (*n* = 80)**	**Healthy volunteer (*n* = 50)** **group I**	**CAD patient (*n* = 30)** **group II**	***p*** **group I vs. group II**
Age [years]	56 ± 17	51 ± 17	64 ± 15	<0.01
Male [*n*]	48 (60%)	25 (50%)	23 (77%)	<0.05
Weight [kg]	77 ± 15	75 ± 13	81 ± 16	<0.05
Height [cm]	172 ± 9	172 ± 9	172 ± 9	n.s.
BMI [kg/m^2^]	26 ± 4	25 ± 3	27 ± 4	<0.05
Systolic blood pressure [mmHg]	124 ± 10	122 ± 8	128 ± 11	<0.05
Diastolic blood pressure [mmHg]	78 ± 7	76 ± 6	80 ± 8	n.s.
Sinus rhythm [*n*]	80 (100%)	50 (100%)	30 (100%)	n.s.
**CV risk factors**				
Hypertension [n]	30 (38%)	7 (14%)	23 (77%)	<0.001
Hypercholesterinemia [n]	23 (29%)	3 (6%)	20 (67%)	<0.001
Diabetes mellitus [n]	7 (9%)	0 (0%)	7 (23%)	<0.001
Family history of CVD [n]	21 (26%)	11 (22%)	10 (33%)	n.s.
Smoker [n]	15 (19%)	3 (6%)	12 (40%)	<0.01
Obesity [n]	23 (29%)	2 (4%)	21 (70%)	<0.001
**Resting CMR parameters**				
Field strength 1.5T [%]	52 (65%)	35 (70%)	17 (57%)	n.s.
LVEF [%]	62 ± 6	63 ± 5	59 ± 6	<0.01
LVEDV [ml]	145 ± 33	142 ± 32	149 ± 34	n.s.
LV mass [g]	103 ± 28	96 ± 27	114 ± 27	<0.01
**Handgrip exercise**				
DHE completed [%]	74 (93%)	48 (96%)	26 (87%)	n.s.
30lb [%]	59 (74%)	36 (72%)	23 (77%)	n.s.
50lb [%]	20 (25%)	13 (26%)	7 (23%)	n.s.
70lb [%]	1 (1%)	1 (2%)	0 (0%)	n.s.

*BMI, body mass index; CVD, cardiovascular disease; T, Tesla; LVEF, left ventricular ejection fraction; LVEDV, left ventricular end-diastolic volume; DHE, dynamic handgrip exercise*.

### Dynamic Handgrip Exercise

Seventy-four persons (93%) fully completed DHE, while a small contingent comprising of six persons (7%) had to abort prematurely due to peripheral fatigue (Table 1). The lightest handgrip ring resistance (30 lb) was used by the majority of subjects (74%), whereas 25% relied on medium resistance (50 lb) and a single person utilized the highest resistance (70 lb).

**Figure 3:**
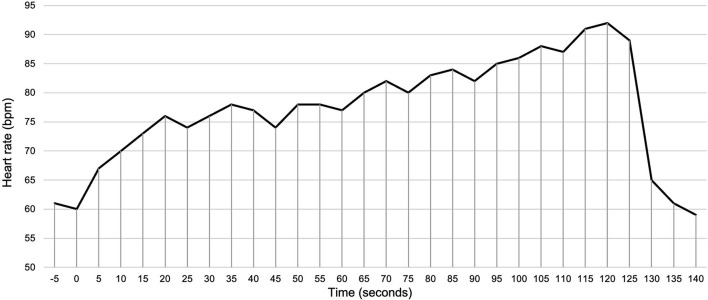
Example of the HR response during DHE of a 29-year-old, male subject. HR increased steadily as DHE progressed. After the end of DHE (120 s), HR fell rapidly toward the resting HR within 10 s. DHE, dynamic handgrip exercise; HR, heart rate.

**Figure 4:**
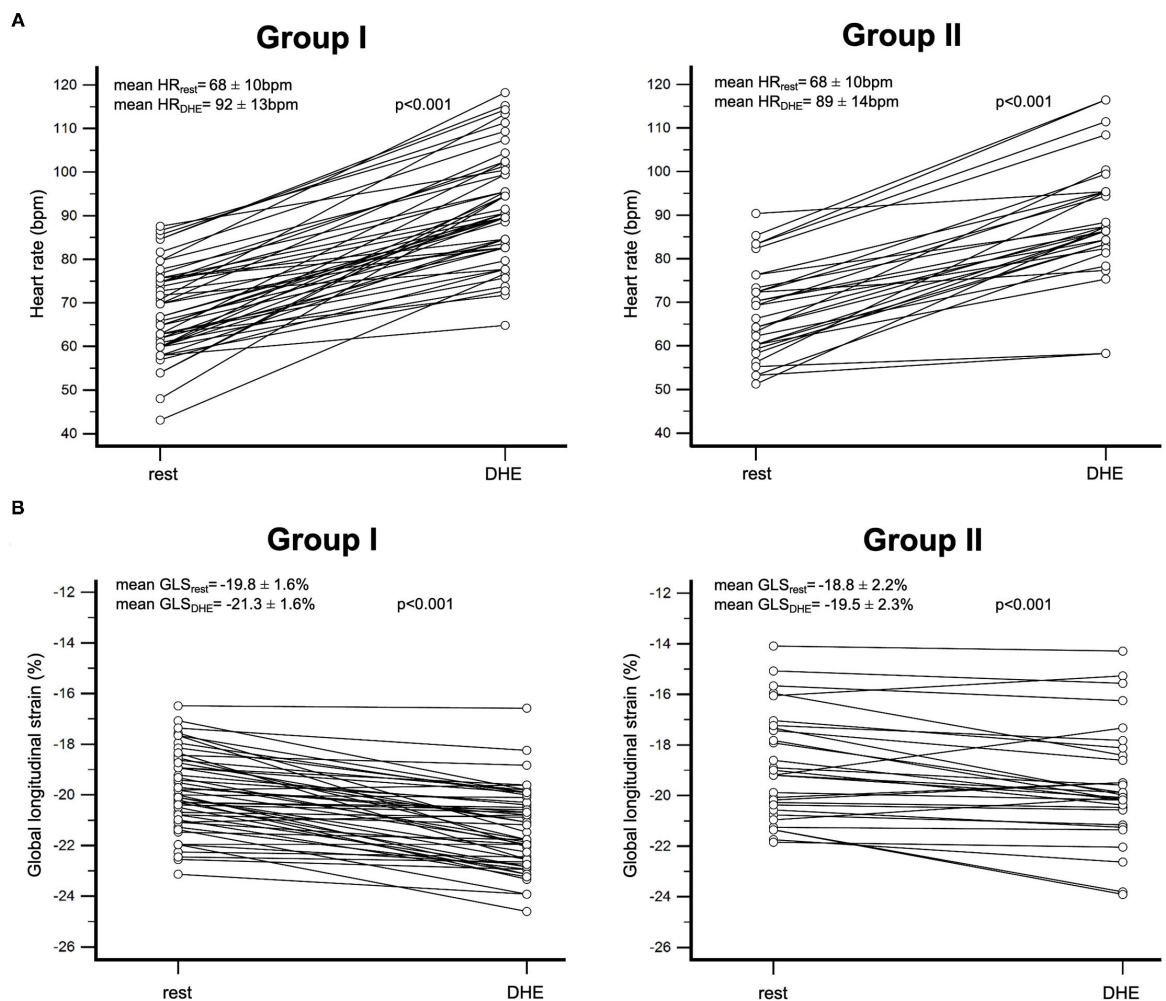
**(A)** Individual response of HR after DHE in healthy individuals (left) and patients with CAD (right). All individuals showed an increase of HR after DHE. Mean HR of all individuals increased from 68 ± 10 bpm at rest to 91 ± 13 bpm after DHE (p < 0.001). **(B)** Individual response of GLS after DHE in healthy individuals (left) and patients with CAD (right). In 70% of our study population, GLS significantly increased (ΔGLS < −0.5%), in 25% it remained unchanged (ΔGLS ≥ −0.5% and ≤ 0.5%) and decreased (ΔGLS > 0.5%) in 5% of patients. Mean GLS increased from −19.4 ± 1.9% at rest to −20.6 ± 2.1% after DHE (p < 0.001). HR, heart rate; LS, longitudinal strain; GLS, global longitudinal strain; DHE, dynamic handgrip exercise; CAD, coronary artery disease.

Mean resting HR was 68 ± 10 bpm. DHE induced a significant increase of HR to 91 ± 13 bpm (*p* < 0.001, Figure 4A). Figure 3 shows a representative course of HR during DHE in a healthy subject. HR increased steadily as DHE progressed. A HR plateau was not evident after 2 min of DHE. After the end of DHE, HR fell rapidly toward the resting HR.

**Figure 5:**
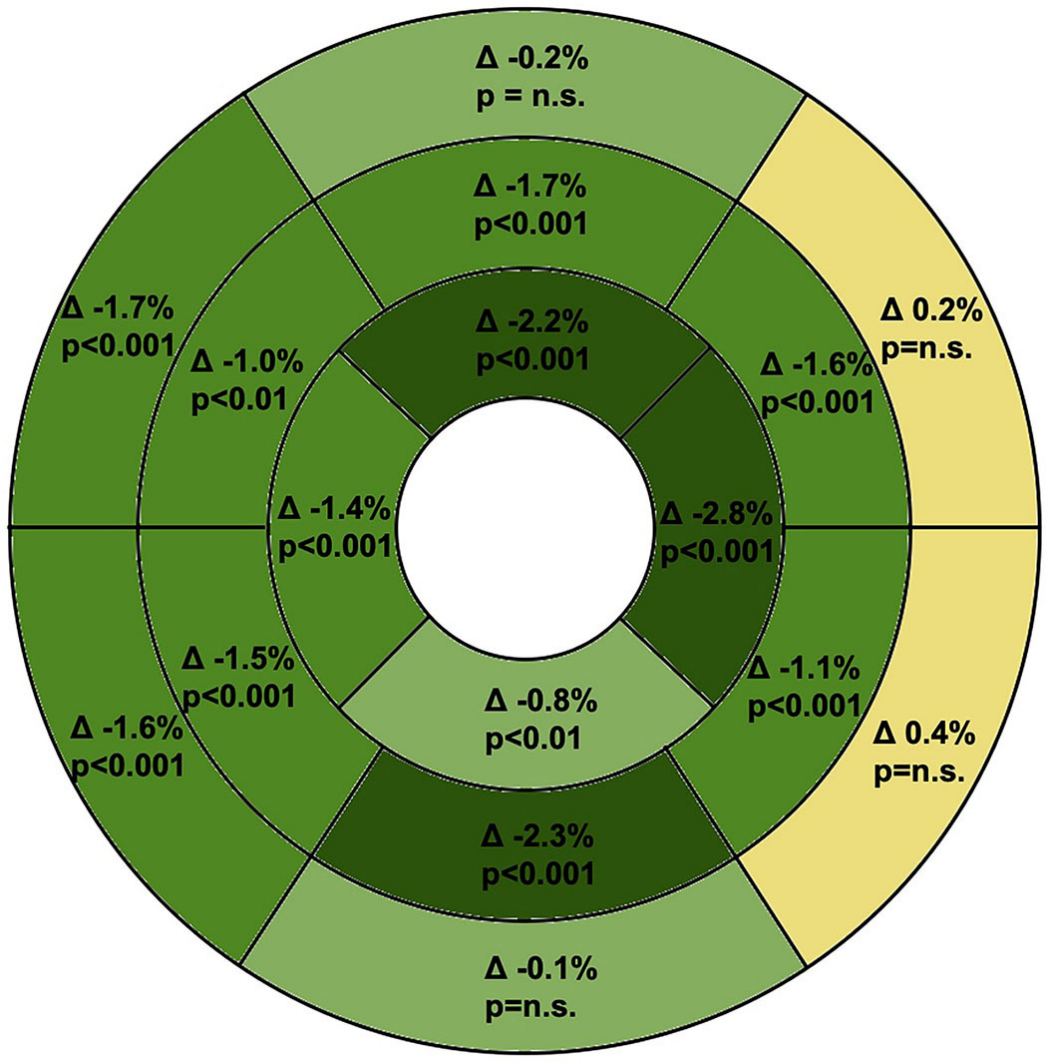
Response of segmental ΔLS after DHE visualized in the AHA 16-Segment model bullseye. LS significantly increased in every midventricular and apical segment. Yellow, ΔLS > 0.0%; bright green, ΔLS 0.0 to –0.9%; green, ΔLS –1.0 to –1.9%; dark green, ΔLS ≤ –2.0%. LS, longitudinal strain; DHE, dynamic handgrip exercise; AHA, American Heart Association.

The GLS at rest was −19.4 ± 1.9%, after DHE we observed an absolute increase of GLS to −20.6 ± 2.1% (*P* < 0.001). The majority of our study population (70%) responded with a relevant increase of GLS (ΔGLS < −0.5%) on a paired comparison, whereas GLS remained unchanged in 25% of the individuals and decreased in 5% (Figure 4B). On a segmental level (Figure 5; Supplementary Tables 1–3), DHE induced a significant increase of LS in every segment of apical and midventricular layer (*p* < 0.01). However, in most basal segments no significant changes of LS could be observed.

**Figure 6:**
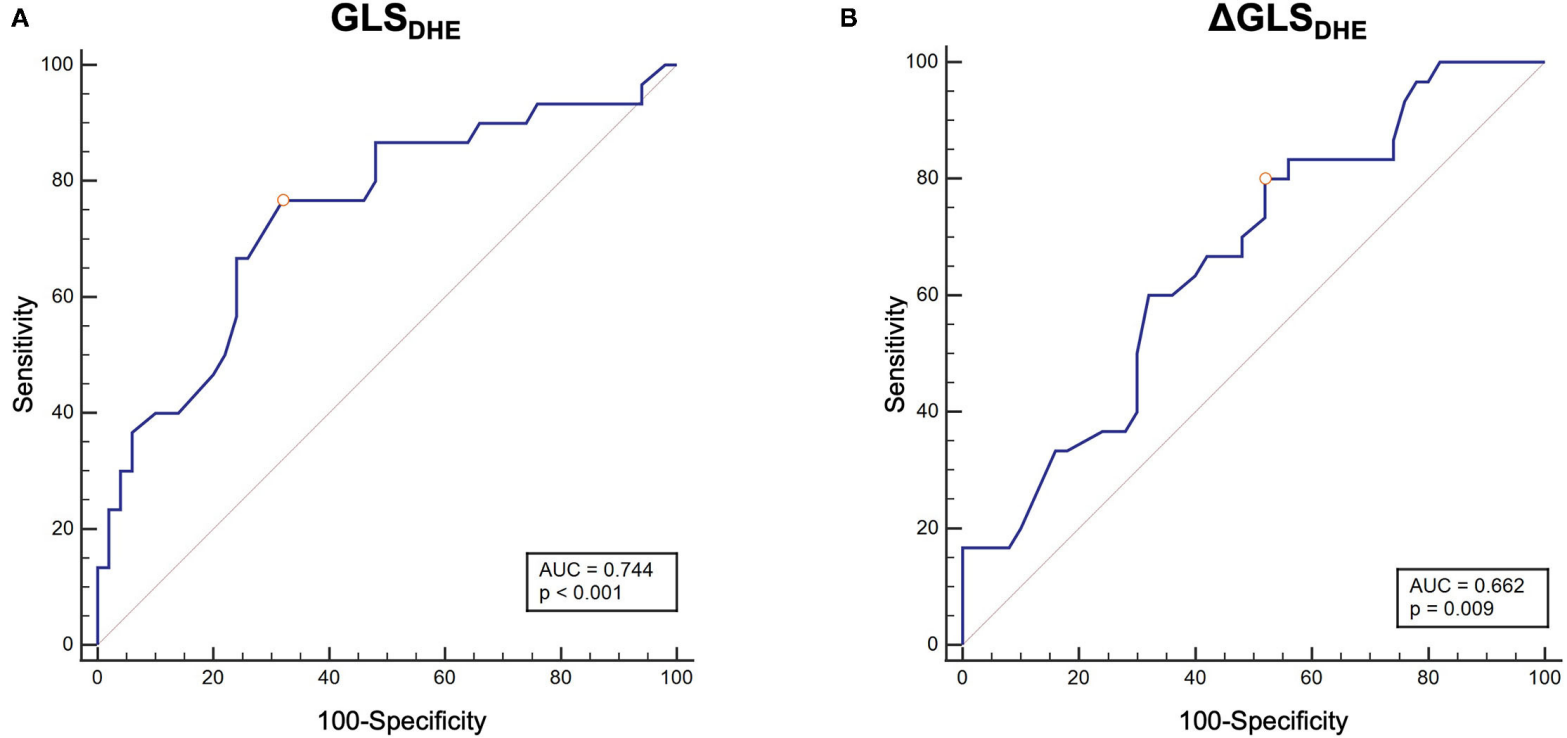
ROC-curve analyses for the differentiation between healthy individuals and patients with CAD. **(A)** GLS_*DHE*_: cut-off value > –20.6%. AUC = 0.744, p < 0.001, sensitivity 76.7%, specificity 68.0%. **(B)** ΔGLS_*DHE*_ (absolute difference between GLS at rest and after DHE): cut-off value >–1.7%. AUC = 0.662, p = 0.009, sensitivity 80.0%, specificity 48.0%. ROC, receiver operating characteristic; CAD, coronary artery disease; GLS, global longitudinal strain; AUC, area under the curve; DHE, dynamic handgrip exercise.

In ROC curve analysis (Figure 6), the absolute change of GLS after DHE (ΔGLS_DHE_) as well as GLS_DHE_ allowed for good differentiation between healthy individuals and patients with CAD using a cut-off value of > −1.7% for ΔGLS_DHE_ (AUC = 0.662, *p* = 0.009, sensitivity 80.0%, specificity of 48.0%) and > −20.6% for GLS_DHE_ (AUC = 0.744, *p* < 0.001, sensitivity 76.7%, specificity of 68.0%).

### Subgroup Analysis of Patients With CAD

Patients in group II (patients with CAD) had a mean age of 64 ± 15 years (23 men, 77%). Four patients (13%) aborted DHE due to peripheral fatigue. Compared to group I, no significant differences were observed for HR at rest and after DHE (*p* = n.s.). In group II, an absolute increase of GLS was observed after DHE (GLS_rest_: −18.8 ± 2.2% vs. GLS_DHE_: −19.5 ± 2.3%, *P* < 0.001). Compared to healthy individuals (group I), the absolute increase of GLS after DHE was significantly lower in group II of patients with CAD (group I: ΔGLS_DHE_ = −1.6 ± 1.3%, group II: ΔGLS_DHE_ = −0.7 ± 1.1%, *p* < 0.01).

**Figure 7:**
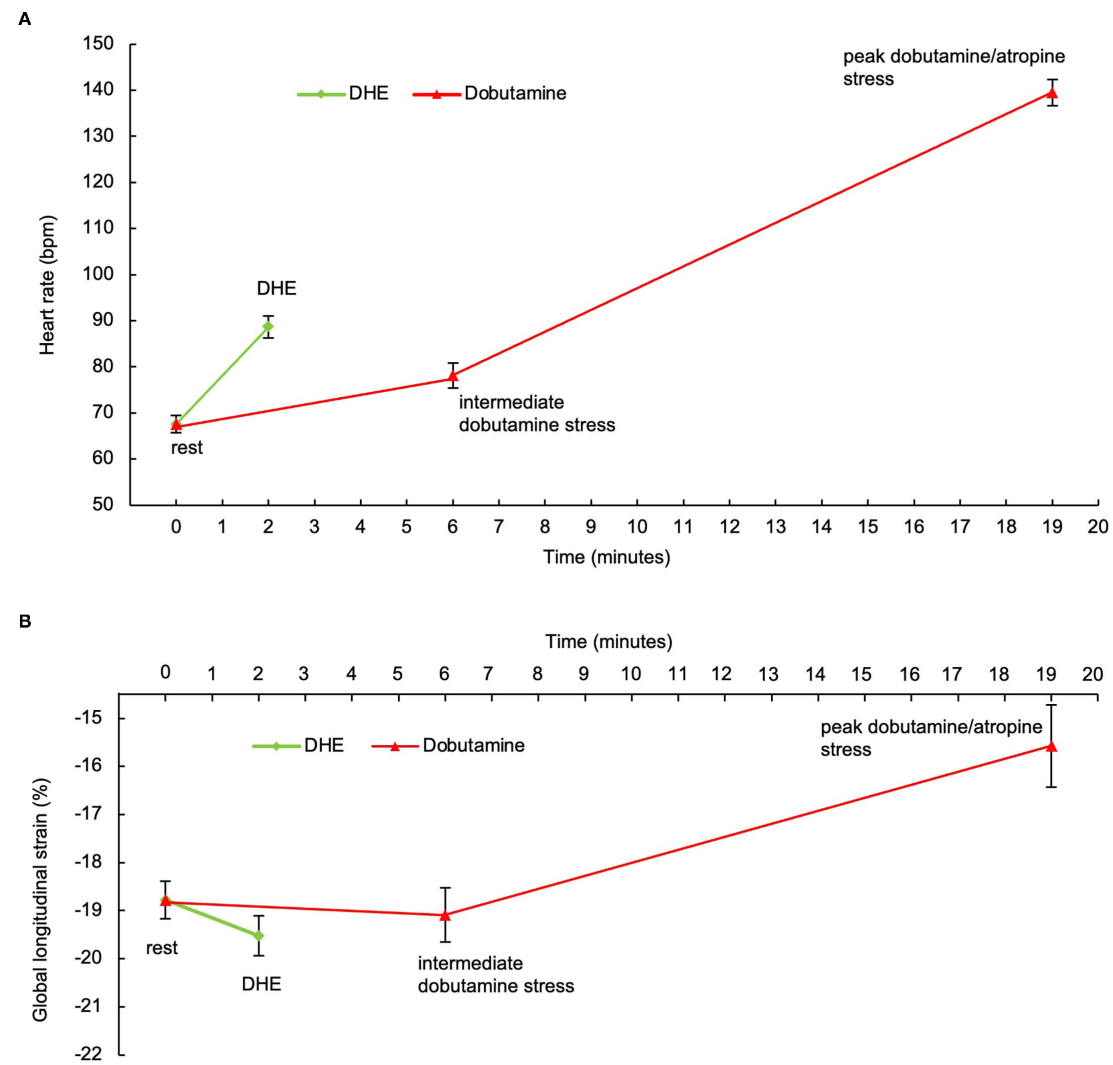
**(A)** Heart response in group II after DHE (green) and during dobutamine stress (red). After 2 min of DHE, HR significantly increased. HR_DHE_ was significantly higher compared with HR during intermediate dobutamine stress. **(B)** GLS response in group II after DHE (green) and during dobutamine stress (red). GLS significantly increased after DHE. During dobutamine stress, GLS increased at intermediate stress level, before it decreased at maximum dobutamine/atropine stress. Error bars represent the standard error of the mean. HR, heart rate; DHE, dynamic handgrip exercise; GLS, global longitudinal strain.

In comparison to intermediate dobutamine stress (Figure 7), a significantly higher HR (HR_DHE_: 89 ± 14 bpm, HR_DobuInterm_: 78 ± 15 bpm, *p* < 0.001) as well as a trend toward a higher GLS was observed after DHE in group II of patients with CAD (GLS_DHE_: −19.5 ± 3.1%, GLS_DobuInterm_: −19.1 ± 3.1%, *p* = 0.22). At peak dobutamine/atropine stress, heart rate was significantly higher compared with DHE and rest (HR_DobuMax_= 140 ± 12 bpm, *p* < 0.001), and GLS though was significantly lower (GLS_DobuMax_= −15.6 ± 3.6%, *p* < 0.001). However, fSENC sequences of 14 patients at maximum stress level (47%) were not able to be evaluated. Physiologic DHE stress including the acquisition of fSENC sequences at rest and after DHE required a median time of 2:20 (2:01–3:23) min. Conversely, intermediate dobutamine stress lasted 6:20 (6:02–6:58) min and maximum dobutamine/atropine stress 19:36 (18:03–22:04) min, which implies a significant time saving of DHE-fSENC (*p* < 0.001).

**Figure 8:**
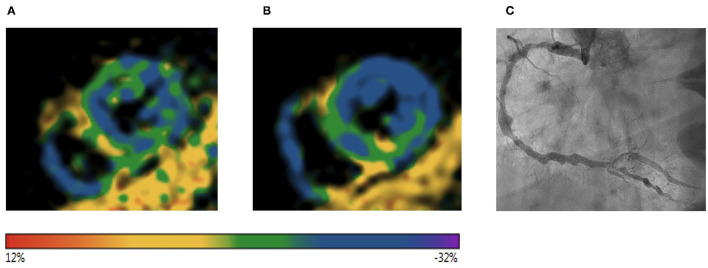
Color-coded fSENC images visualizing longitudinal strain at rest **(A)** and after DHE **(B)** of an excluded patient with a positive dobutamine stress test. Invasive coronary angiography revealed a significant stenosis of the right coronary artery **(C)** (color scale represents regional longitudinal strain). fSENC, fast strain-encoded magnetic resonance imaging; DHE, dynamic handgrip exercise.

In total, five patients with CAD were excluded from our final study cohort due to a positive dobutamine stress CMR: 13 segments showed wall motion abnormalities as an indicator of myocardial ischemia. In the fSENC analysis during intermediate dobutamine stress, longitudinal strain of the ischemic segments showed an absolute decrease (−19.5 ± 3.2% to −16.7 ± 3.2%, *p* < 0.001). At peak dobutamine stress, LS worsened (−19.5 ± 3.2% to −15.0 ± 1.2%, *p* < 0.01), while it should be noted that fSENC in two patients was not able to be analyzed during peak dobutamine stress. After DHE, an absolute decrease of GLS was observed in the corresponding “ischemic” segments (−19.5 ± 3.2% to −16.2 ± 2.5%, *p* < 0.001). Subendocardial worsening of LS after DHE (B) is illustrated in Figure 8 showing a patient with significant stenosis of the right coronary artery (C).

### Subgroup Analysis of Group I (Healthy Individuals) for Gender and Age Differences

Gender-related subgroup analysis revealed no significant differences regarding LVEF (*p* = n.s.). Significantly more women used the lowest (30 lb) handgrip ring (24 women (96.0%) vs. 12 men (48.0%), *p* < 0.001). No significant gender differences were found for HR_rest_ and HR_DHE_. In contrast, GLS_rest_ (men: −19.0 ± 1.3%, women: −20.5 ± 1.5%, *p* < 0.001) and GLS_DHE_ (men: −20.5 ± 1.5%, women: −21.9 ± 1.3%, *p* < 0.05), but not ΔGLS (men: −1.7 ± 1.1%, women: −1.3 ± 1.4%, *p* = n.s.) were significantly different between male and female subjects.

Divided at the median age (53.7 years), two age-dependent groups were created: young (*n* = 25, mean age = 36.8 ± 10.4 years) and old adults (*n* = 25, mean age = 65 ± 7 years). Except HR_DHE_ (young HR_DHE_ = 96 ± 12bpm vs. old HR_DHE_ = 88 ± 12bpm; *p* < 0.05), no significant differences were observed between young and old adults related to DHE study. Neither age, gender, or handgrip ring strength were significant confounders for heart rate or GLS stress response.

### Observer Variability

Quantification of both GLS_rest_ and GLS_DHE_ by fSENC featured an excellent reproducibility. ICC for the intraobserver variability of GLS_rest_ was 0.98 (95% CI: 0.93–1.00) and for the interobserver variability it was 0.98 (95% CI: 0.95–1.00). For GLS_DHE_, the ICC for intraobserver variability was 0.99 (95% CI: 0.89–1.00) and for interobserver variability it was 0.97 (95% CI: 0.88–0.99).

## Discussion

In our study, we examined the feasibility of DHE and its hemodynamic effects. We observed an absolute increase of GLS and heart rate after DHE, which implies a positive chrono- and inotropic response. The effect strength of DHE is non-inferior to intermediate dobutamine-stimulation. GLS_DHE_ allowed for suitable differentiation between healthy individuals and patients with CAD. Furthermore, the reasonable low DHE abortion rate supports the practicability of the approach.

As a potential alternative to conventional stress testing, exercise-CMR allows for a needle-free protocol without pharmacological side effects. Due to its simple and favorable handling, DHE represents a cost- and timesaving physiologic stressor. Although, the hemodynamic impact of DHE does not achieve standard target heart rate criteria (≥85% of maximum heart rate), strain imaging had demonstrated in the past to allow the detection of ischemia at intermediate dobutamine-stimulation with high accuracy using Strain-ENCoded CMR (20). As shown in our group of patients with CAD, DHE achieved a comparable or slightly higher increase of HR and longitudinal strain than intermediate dobutamine stress. Due to its similar chronotropic and inotropic effects, DHE represents a promising, needle-free stressor to induce ischemia. In future, prospective trials in patients with CAD should determine the potential of DHE-fSENC for the detection of obstructive CAD.

To detect segmental stress-induced functional impairment, fSENC appears to fulfill the prerequisites of a fast and reliable strain assessment, and other techniques, like feature tracking, are known to struggle with segmental reproducibility, and myocardial tagging is very time consuming in both preparation and acquisition (15, 18). As recently shown by our group, measurements of segmental longitudinal strain using fSENC allows for the detection of ischemia-related wall motion abnormalities after hyperventilation/breath-hold maneuver and during adenosine stress (21). As our data show, the response of GLS after DHE allows for the precise detection of CAD. The analysis of the “excluded” patients due to a positive dobutamine stress showed the potential to detect obstructive CAD as a needle-free CMR stress test. Furthermore, a predecessor technology, SENC, has already been shown to identify patients at risk for future cardiac events and revascularization during dobutamine stress CMR (22).

Remarkably, our data suggest a higher chronotropic effect using repetitive, two-handed exercise compared with other studies using different variations of handgrip exercise (19). In contrast, previous studies had evaluated isometric one-handed handgrip exercise protocols with a broad range of exercise duration, handgrip application, and devices hampering a general comparability (19, 23–25). However, several comparative studies investigated the differences between dynamic and isometric (static) handgrip exercise (26–29). Although most authors found no significant differences in hemodynamic response, Stebbins et al. observed a significantly higher increase of heart rate, blood pressure, and cardiac output with increasing handgrip strengths compared with isometric handgrip protocols. In comparison to intermediate dobutamine stimulation, DHE achieved higher heart rates reflecting a better chronotropic response. In contrast to an isolated increase of afterload as observed in isometric handgrip exercise, DHE increases the production and accumulation of muscle metabolites and may consequently lead to a higher exercise pressor reflex and greater activation of muscle mechanoreceptors (29).

Regarding the GLS response after handgrip exercise, several authors made different observations (19, 24, 30, 31). Most recently, Blum et al. examined the response of GLS after an isometric handgrip exercise using fSENC-CMR, as we did (19). They assumed a limited diagnostic purpose for strain imaging after isometric handgrip exercise due to a heterogenous response of GLS. To our knowledge, data on the GLS response after DHE do not exist so far. In our study population, GLS significantly increased after DHE. The vast majority of 56 subjects (70.0%) had a relevant increase of < -0.5%. Even on a segmental level, the LS increased significantly in most segments, which is important for its potential future use in detecting regional impairment in ischemic myocardium. In comparison to isometric handgrip exercise, our results are more homogenous and the LS increased significantly in the vast majority of patients suggesting that DHE could be a more suitable protocol for future use with fSENC-CMR.

### Limitations

In the present study we did not investigate varying durations of DHE and their hemodynamic impact. A prolongation of the stress appears to be of potential benefit if tolerated by the patient, as heart rates did not reach a plateau level. Moreover, continuous heart rate and blood pressure monitoring was not available during stress testing. The non-invasive blood pressure monitoring with a common upper arm cuff did not allow for reliable measurements during the repetitive contractions. Peak exercise blood pressure would have allowed to better understand the development of left ventricular afterload. Due to a worsening of the ECG signal, fSENC sequences had to be started after breath-hold and after finishing DHE, which might influence the amount of longitudinal strain. Measurements of longitudinal strain were acquired at 1.5 T and 3 T scanners. So far, no data exist regarding the comparability of fSENC data acquired at different magnetic field strengths.

The number of ischemic cases in group II of patients with CAD was too low to allow an analysis of diagnostic accuracy for the detection of ischemia. The prospective study was not powered for this question.

## Conclusion

Dynamic handgrip exercise causes a positive inotropic and chronotropic effect comparable or slightly higher than intermediate dobutamine stress, suggesting a relevant increase of myocardial oxygen demand. In this rather small cohort, DHE appears safe with broad applicability. Even minor differences of longitudinal strain can be detected fast and reliably using CMR-fSENC. Further studies which investigate the differences of isometric and DHE, and also the influence of one- and two-handed approaches with regard to the response of LS are needed. Nevertheless, the data encourages further studies to determine its potential to detect obstructive CAD as a potential needle-free CMR stress test.

## Data Availability Statement

The raw data supporting the conclusions of this article will be made available by the authors, without undue reservation.

## Ethics Statement

The studies involving human participants were reviewed and approved by Ethics Committee of the University of Heidelberg (medical faculty). The patients/participants provided their written informed consent to participate in this study. Written informed consent was obtained from the individual(s) for the publication of any potentially identifiable images or data included in this article.

## Author Contributions

AO, HK, NF, MF, and MO were responsible for conception of study design. AO, MN, JR, JS, LW, FA, CS, NO, EG, MM-H, and MO acquired and analyzed the data. AO, MN, and MO made the statistical analysis. AO drafted the manuscript. All authors interpreted the data and contributed to the revision of the manuscript and finally approved the latest version.

## Conflict of Interest

NO is the CSO and founder of Myocardial Solutions, provider of MyoStrain^®^ software for the analysis of fSENC sequences. CS is an employee at Philips Healthcare. The remaining authors declare that the research was conducted in the absence of any commercial or financial relationships that could be construed as a potential conflict of interest.

## Publisher's Note

All claims expressed in this article are solely those of the authors and do not necessarily represent those of their affiliated organizations, or those of the publisher, the editors and the reviewers. Any product that may be evaluated in this article, or claim that may be made by its manufacturer, is not guaranteed or endorsed by the publisher.

## Abbreviations

bpm: beats per minute
CAD: coronary artery disease
CMR: cardiac magnetic resonance
DHE: dynamic handgrip exercise
EF: ejection fraction
fSENC: fast strain-encoded magnetic resonance imaging
GLS: global longitudinal strain
HR: heat rate
LS: longitudinal strain
LV: left ventricular
MVC: maximum voluntary contraction.
