# Supplementary material for: Dynamic Handgrip Exercise: Feasibility and Physiologic Stress Response of a Potential Needle-Free Cardiac Magnetic Resonance Stress Test
Source: Front Cardiovasc Med. 2021 Nov 29;8:755759. doi: 10.3389/fcvm.2021.755759 (PMC8666587; doi:10.3389/fcvm.2021.755759)
Supplement: Supplementary file 1 [file Table_1.docx]

***Table 1*** **Comparison of segmental longitudinal strain at rest and after DHE in the main group (n=80)**

DHE= dynamic handgrip exercise; GLS= global longitudinal strain.

|  | **GLS_rest_** | **GLS_DHE_** | **ΔGLS_DHE_** | **p** |
| --- | --- | --- | --- | --- |
| **basal anterior** | -18.3 ± 4.2 | -18.5 ± 5.1 | -0.2 ± 4.0 | n.s. |
| **basal anteroseptal** | -13.7 ± 4.7 | -15.5 ± 5.0 | -1.7 ± 3.5 | < 0.001 |
| **basal inferoseptal** | -19.6 ± 4.7 | -21.3 ± 4.3 | -1.6 ± 4.2 | < 0.001 |
| **basal inferior** | -23.7 ± 3.1 | -23.7 ± 2.9 | -0.1 ± 3.0 | n.s. |
| **basal inferolateral** | -24.7 ± 2.5 | -24.3 ± 2.8 | 0.4 ± 3.0 | n.s. |
| **basal anterolateral** | -23.0 ± 3.3 | -22.9 ± 3.8 | 0.2 ± 3.6 | n.s. |
| **midventricular anterior** | -17.9 ± 3.2 | -19.6 ± 3.6 | -1.7 ± 2.7 | < 0.001 |
| **midventricular anteroseptal** | -18.6 ± 3.6 | -19.5 ± 3.7 | -1.0 ± 2.9 | < 0.01 |
| **midventricular inferoseptal** | -18.5 ± 3.8 | -20.1 ± 4.2 | -1.5 ± 3.1 | < 0.001 |
| **midventricular inferior** | -18.6 ± 3.3 | -20.9 ± 3.8 | -2.3 ± 3.5 | < 0.001 |
| **midventricular inferolateral** | -21.0 ± 2.8 | -22.1 ± 3.2 | -1.1 ± 2.7 | < 0.001 |
| **midventricular anterolateral** | -19.3 ± 2.9 | -20.9 ± 3.4 | -1.6 ± 2.8 | < 0.001 |
| **apical anterior** | -15.6 ± 4.0 | -17.8 ± 4.0 | -2.2 ± 3.3 | < 0.001 |
| **apical septal** | -21.4 ± 3.9 | -22.7 ± 3.6 | -1.4 ± 2.7 | < 0.001 |
| **apical inferior** | -20.7 ± 3.3 | -21.5 ± 3.5 | -0.8 ± 2.4 | <0.01 |
| **apical lateral** | -16.4 ± 3.9 | -18.9 ± 3.7 | -2.8 ± 2.6 | < 0.001 |
| **global** | **-19.4 ± 1.9** | **-20.6 ± 2.1** | **-1.2 ± 0.9** | **< 0.001** |

***Table 2*** **Comparison of segmental longitudinal strain at rest and after DHE in group I of healthy individuals (n=50)**

DHE= dynamic handgrip exercise; GLS= global longitudinal strain.

|  | **GLS_rest_** | **GLS_DHE_** | **ΔGLS_DHE_** | **p** |
| --- | --- | --- | --- | --- |
| **basal anterior** | -19.3 ± 3.5 | -20.1 ± 4.4 | -0.9 ± 3.5 | n.s. |
| **basal anteroseptal** | -14.8 ± 4.3 | -17.0 ± 3.9 | -2.2 ± 3.4 | < 0.01 |
| **basal inferoseptal** | -20.5 ± 4.5 | -22.0 ± 4.0 | -1.4 ± 3.9 | n.s. |
| **basal inferior** | -24.3 ± 3.0 | -24.1 ± 2.8 | 0.3 ± 3.3 | n.s. |
| **basal inferolateral** | -25.0 ± 2.4 | -24.3 ± 2.9 | 0.7 ± 3.3 | n.s. |
| **basal anterolateral** | -23.5 ± 3.4 | -23.4 ± 4.0 | 0.1 ± 3.8 | n.s. |
| **midventricular anterior** | -18.1 ± 3.1 | -20.1 ± 3.4 | -2.0 ± 3.0 | < 0.01 |
| **midventricular anteroseptal** | -18.8 ± 2.9 | -20.2 ± 3.3 | -1.3 ± 2.7 | < 0.05 |
| **midventricular inferoseptal** | -18.7 ± 3.6 | -20.6 ± 3.9 | -1.9 ± 2.8 | < 0.05 |
| **midventricular inferior** | -18.8 ± 3.2 | -21.6 ± 3.1 | -2.8 ± 3.7 | < 0.001 |
| **midventricular inferolateral** | -21.2 ± 2.7 | -22.8 ± 2.8 | -1.5 ± 2.3 | < 0.001 |
| **midventricular anterolateral** | -19.4 ± 2.9 | -21.2 ± 3.2 | -1.8 ± 2.6 | < 0.01 |
| **apical anterior** | -15.9 ± 4.2 | -18.6 ± 3.5 | -2.7 ± 2.7 | < 0.001 |
| **apical septal** | -22.0 ± 3.2 | -23.4 ± 2.7 | -1.4 ± 2.5 | < 0.05 |
| **apical inferior** | -21.3 ± 3.0 | -22.3 ± 3.2 | -1.0 ± 2.3 | n.s. |
| **apical lateral** | -16.7 ± 3.7 | -19.6 ± 3.6 | -2.8 ± 2.6 | < 0.01 |
| **global** | **-19.8 ± 1.6** | **-21.3 ± 1.6** | **-1.6 ± 1.3** | **< 0.001** |

***Table 3*** **Comparison of segmental longitudinal strain at rest and after DHE in group II of CAD patients (n=30)**

DHE= dynamic handgrip exercise; GLS= global longitudinal strain.

|  | **GLS_rest_** | **GLS_DHE_** | **ΔGLS_DHE_** | **p** |
| --- | --- | --- | --- | --- |
| **basal anterior** | -16.6 ± 4.7 | -15.8 ± 4.9 | 0.8 ± 4.5 | n.s. |
| **basal anteroseptal** | -12.1 ± 4.9 | -13.0 ± 5.4 | -0.9 ± 3.4 | n.s. |
| **basal inferoseptal** | -18.2 ± 4.8 | -20.1 ± 4.6 | -1.9 ± 4.5 | < 0.05 |
| **basal inferior** | -22.6 ± 3.1 | -23.2 ± 2.9 | -0.6 ± 2.3 | n.s. |
| **basal inferolateral** | -24.4 ± 2.4 | - 24.4 ± 2.6 | -0.01 ± 2.5 | n.s. |
| **basal anterolateral** | -22.3 ± 3.1 | -22.0 ± 3.2 | 0.2 ± 3.3 | n.s. |
| **midventricular anterior** | -17.6 ± 3.3 | - 18.7 ± 3.9 | -1.2 ± 2.1 | < 0.01 |
| **midventricular anteroseptal** | -18.1 ± 4.5 | -18.5 ± 4.1 | -0.3 ± 3.1 | n.s. |
| **midventricular inferoseptal** | -18.4 ± 4.3 | - 19.2 ± 4.5 | -0.8 ± 3.5 | n.s. |
| **midventricular inferior** | -18.6 ± 3.6 | -19.8 ± 4.4 | -1.3 ± 3.1 | < 0.05 |
| **midventricular inferolateral** | -20.9 ± 3.1 | -21.1 ± 3.5 | -0.2 ± 3.2 | n.s. |
| **midventricular anterolateral** | -19.2 ± 3.1 | -20.5 ± 3.5 | -1.2 ± 3.2 | < 0.05 |
| **apical anterior** | -15.2 ± 3.6 | -16.5 ± 4.4 | -1.0 ± 3.6 | n.s. |
| **apical septal** | -20.4 ± 4.6 | -21.7 ± 4.6 | -1.3 ± 3.0 | < 0.05 |
| **apical inferior** | -19.9 ± 3.5 | -20.4 ± 3.5 | -0.6 ± 2.5 | n.s. |
| **apical lateral** | -16.0 ± 4.2 | -18.0 ± 3.7 | -1.9 ± 2.2 | < 0.001 |
| **global** | **-18.8 ± 2.2** | **-19.5 ± 2.3** | **-0.7 ± 1.1** | **< 0.001** |
